# Improving accuracy in state of health estimation for lithium batteries using gradient-based optimization: Case study in electric vehicle applications

**DOI:** 10.1371/journal.pone.0293753

**Published:** 2023-11-02

**Authors:** Mouncef El Marghichi, Soufiane Dangoury, Younes zahrou, Azeddine Loulijat, Hamid Chojaa, Fahd A. Banakhr, Mohamed I. Mosaad

**Affiliations:** 1 Faculty of Sciences and Technology, Hassan First University, Settat, Morocco; 2 Hassan II University of Casablanca, Casablanca, Morocco; 3 Higher Normal School of Technical Education Mohammedia, Mohammedia, Morocco; 4 Industrial Technologies and Services Laboratory, Higher School of Technology, Sidi Mohamed Ben Abdellah University, Fez city, Morocco; 5 Electrical & Electronics Engineering Technology Department, Yanbu Industrial College (YIC), Royal Commission Yanbu Colleges & Institutes, Yanbu, Saudi Arabia; 6 Faculty of Engineering, Department of Electrical Engineering, Damietta University, Damietta, Egypt; Vellore Institute of Technology, INDIA

## Abstract

Significant improvements in battery performance, cost reduction, and energy density have been made since the advancements of lithium-ion batteries. These advancements have accelerated the development of electric vehicles (EVs). The safety and effectiveness of EVs depend on accurate measurement and prediction of the state of health (SOH) of lithium-ion batteries; however, this process is uncertain. In this study, our primary goal is to enhance the accuracy of SOH estimation by reducing uncertainties in state of charge (SOC) estimation and measurements. To achieve this, we propose a novel method that utilizes the gradient-based optimizer (GBO) to evaluate the SOH of lithium batteries. The GBO minimizes a cost with the aim of selecting the optimal candidate for updating the SOH through a memory-fading forgetting factor. We evaluated our method against four robust algorithms, namely particle swarm optimization-least square support vector regression (PSO-LSSV), BCRLS-multiple weighted dual extended Kalman filtering (BCRLS-MWDEKF), Total least square (TLS), and approximate weighted total least squares (AWTLS) in hybrid electric vehicle (HEV) and electric vehicle (EV) applications. Our method consistently outperformed the alternatives, with the GBO achieving the lowest maximum error. In EV scenarios, GBO exhibited maximum errors ranging from 0.65% to 1.57% and mean errors ranging from 0.21% to 0.57%. Similarly, in HEV scenarios, GBO demonstrated maximum errors ranging from 0.81% to 3.21% and mean errors ranging from 0.39% to 1.03%. Furthermore, our method showcased superior predictive performance, with low values for mean squared error (MSE) (<1.8130e-04), root mean squared error (RMSE) (<1.35%), and mean absolute percentage error (MAPE) (<1.4).

## 1. Introduction

Electric vehicles are widely recognized as a crucial solution to combat environmental pollution and tackle energy shortages. With the ability to use various sources of energy and multiple modes, EVs can conserve energy and improve the environment [1–3]. Lithium-ion batteries are widely used as power sources in EVs due to their efficiency. However, their performance degrades over time due to aging, which reduces their energy storage and power delivery capacity. Therefore, evaluating the state of health (SOH) of the battery is crucial to ensure a safe and enjoyable driving experience [4]. Once the SOH of the battery reaches the preset limit, it must be replaced with a new one to maintain optimal EV performance.

The most commonly accepted metrics regarding SOH include cell capacity and battery internal resistance, reflecting the energetic capacity and power capacity, respectively [5,6]. Based on the survey of internal ageing strategies of different kinds of batteries, the leakage of battery lithium storage, breakdown of effective materials, and physical alterations are the most popular reasons for the capacity loss, the resistance rise is primarily caused by the solid electrolyte interface (SEI) layer growth [7–9]. The battery testing procedure manuals state that when the battery capacity falls below 80% of the original nominal rating within a particular test procedure, the battery is considered unsuitable for the vehicle application and should be replaced [10]. However, in some instances, the increase in internal resistance leading to a decrease in power will obviously lead to the failure of the battery in advance. Therefore, it is crucial to consider these two aspects combined for EVs to function normally. Battery characteristics that change significantly during ageing may also point to the growing potential for battery failure, like charge curves, OCV (open circuit voltage) charts, etc. Many tests have been performed in the laboratory to disclose the ageing pattern of such health-based metrics [11,12].

Various approaches to assess the SOH of the batteries have been developed, and they may be further broken down into the main following groups: Physical models, empiric models, incremental differential voltage/current analysis (ICA/DVA), and data-based techniques [7,8]. The physical methods rely upon mathematically based models describing the internal reaction characteristics of the battery within the electrodes and on their surfaces. They have high precision [9], yet they often encounter significant difficulties in practical implementations due to the model complexity and the identification of the parameters [10]. The empirical models utilize adaptive algorithms that use empirical battery models like the equivalent circuit model [13,14] and the electrochemically reduced model [8]. Such models can be readily implemented for online SOH surveillance, but the development of the algorithm often needs extensive experiment testing and debugging to achieve proper precision [8]. An alternative approach to empirical modeling is to utilize a small set of preset experimental data to enhance the model, but their precision is restricted to conditions similar to those of the experimental data [15].

As battery technology advances, data-driven methods are receiving increased attention thanks to their model-free features [16]. These techniques map out the external properties of the battery to its current capacity (SOH) drop, or using previous capacities and global degradation as input components. Numerous methods have been developed using data-driven methods, such as: Support vector machines [17,18], Bayesian networks [19,20], autoregressive models [21], and Gaussian process regression (GPR) [22]. Despite their nonlinear properties and high estimation accuracy, data-driven methods need access to top-quality data sets for training purposes. For example, Xie et al [23] used a deep learning-based method for estimating the SOH of a lithium-ion battery using a dataset that included multiple battery types and operating conditions. The results showed that the proposed method had high accuracy in estimating the battery SOH but required large amounts of high-quality data for training. Similarly, the paper [24] proposed a hybrid approach that combined data-driven and physics-based models to estimate the SOH of a lithium-ion battery. The method achieved high accuracy in estimating the battery SOH but required top quality data for training and validation.

The paper in [25] employs data-driven methods and introduces an aging feature extraction technique based on an electrochemical model (EM). This method extracts internal health features (IHFs) associated with battery degradation, such as charge transfer resistance and solid phase diffusion coefficient. External health features (EHFs) are also derived from voltage and temperature curves. The selected IHFs and multi-stage EHFs are used to enhance SOH estimation using machine learning algorithms, demonstrating improved accuracy across various scenarios and battery charge-discharge modes. This approach bridges the gap between physical and data-driven models while boosting SOH estimation precision. The study [26] introduces an improved radial basis function neural network (IRBFNN). Unlike traditional neural networks, this IRBFNN combines a linear polynomial to capture long-term battery capacity trends and non-linear transformations in the hidden layers for local capacity changes. The network parameters are optimized using an improved gray wolf optimization (IGWO) algorithm. Four features extracted during partial constant current charging are used as inputs. Experimental results on NASA and CALCE datasets demonstrate that the IRBFNN-based method accurately estimates SOH with maximum errors within ±4%. In contrast, the paper [27] employs a combination of data preprocessing techniques and a CNN-Transformer framework. The data preprocessing steps involve feature selection using the Pearson correlation coefficient (PCC), the application of principal component analysis (PCA) to reduce computational complexity, and feature scaling through min-max normalization for faster training. Subsequently, the CNN-Transformer model is applied to the preprocessed data, and training and testing are conducted using the NASA battery dataset. The results underscore the effectiveness of this approach, with absolute estimation errors staying within 1% and performance metrics like mean absolute error (MAE), mean absolute percentage error (MAPE), and root mean square error (RMSE) all constrained within 0.55%. By harnessing the strengths of both CNNs and Transformers, this model proves proficient in capturing long-term dependencies and providing precise and stable SOH estimations for lithium-ion batteries. The authors in [28] developed a novel hybrid neural network model with attention mechanisms. The model, named CNN-CBAM-LSTM, combines convolution neural network (CNN), convolutional block attention module (CBAM), and long short-term memory (LSTM) neural network components. This innovative approach leverages partial charging voltage curves as direct inputs, achieving high-accuracy SOH estimation. The CBAM’s sequential attention structure enhances CNN’s ability to extract relevant battery health features while reducing the impact of noise in raw data. Additionally, the study demonstrates the model’s effectiveness under various operational conditions and data sampling modes. Transfer learning with fine-tuning is employed to adapt the model to different battery operating scenarios. Validation on two public datasets yields impressive results, with root mean square errors and mean absolute errors for the best estimates at 0.17% and 0.14%, respectively.

One major challenge facing data-driven models for estimating the SOH in practical conditions is the need for training data that includes information on battery capacity and health features. However, obtaining such data can be difficult in electric vehicles (EVs) as the battery is rarely fully cycled and its capacity status is often unknown. This lack of available data can limit the accuracy and effectiveness of data-driven approaches for estimating SOH in real-world scenarios. Overall, while data-driven methods have shown promising results in estimating the battery SOH, careful consideration must be given to the quality and quantity of data used for training and validation.

Most methods for estimating the capacity and, in turn, the battery SOH rely on the following equation:

$$\underset{y_{j}}{\underset{︸}{\int_{t0}^{t1}\eta i(t)dt}}=Q\times \underset{x_{j}}{\underset{︸}{\left(SOC(t_{1})‐SOC(t_{0}\right)}}$$ (1)

Where *Q* refers to the battery capacity, and *i(t)* is the battery current, *η* is the efficiency factor.

The linear structure of *y*_*j*_ *= Qx*_*j*_ is clear, and *Q* can be estimated using a regression technique. The only requirement is to determine values for "*x*_*j*_" and "*y*_*j*_". The challenge arises from the presence of noise in *y*_*j*_ and *x*_*j*_. Therefore, the equation becomes: *(y*_*j*_ *-Δ y*_*j*_*) = Q(x*_*j*_ *-Δx*_*j*_*)*. Neglecting these uncertainties can lead to inaccurate SOH estimates and, consequently, affect the battery’s overall performance. Therefore, it is crucial to consider both sources of noise when estimating the SOH of a battery.

The conventional linear regression technique (least squares) is problematic as both the accumulated current *y*_*j*_ and the difference in the state of charge (SOC) values *x*_*j*_ are prone to noise from sensors or estimation. This approach assumes that only the measurements *y*_*j*_ have noise, but the independent variable *x*_*j*_ is noise-free. However, in reality, both the SOC estimates and integrated current have noise. As a result, using the standard least squares linear regression method yields a biased and inaccurate evaluation of battery capacity because of the noise on the *x*_*j*_ variable [29].

Plett’s work on minimizing noise in battery capacity estimation was considered a major contribution, which was documented in publications [29,30]. Plett proposed four least square algorithms that could estimate battery capacity, and consequently, SOH while taking into account noise on both the *x*_*j*_ and *y*_*j*_ axes. In the same vein, in previous research [31], we employed the Sunflower Optimization Algorithm, which has shown promising results in achieving the same goal. Our study found that the Sunflower Optimization Algorithm could estimate battery capacity (SOH) while accounting for noise, resulting in accurate and unbiased estimates of battery capacity.

Paper [32] focuses on enhancing battery capacity estimation (SOH) accuracy by introducing an innovative approach leveraging the bald eagle search algorithm (BES) to mitigate uncertainties in state of charge (SOC) estimation. BES strategically navigates the search space, minimizing a designated cost function, and incorporates a memory-forgetting factor for real-time cell capacity updates. The study’s distinctiveness lies in integrating BES into battery capacity optimization and including a memory-forgetting factor. Validation with NASA’s Prognostic Data and various battery scenarios demonstrates BES’s consistent outperformance of four aggressive algorithms, with a peak error rate of only 1.06%. This approach also highlights the importance of reducing measurement noise and SOC prediction errors, suggesting using sigma-point Kalman filters (SPKF) to enhance SOC estimation precision while avoiding circular dependencies. The study presented in [33] is primarily concerned with achieving precise battery capacity (SOH) estimation, which is crucial for efficient battery management systems, especially in predicting potential failures via the state of health (SOH) metric. This research introduces a novel algorithm, Enhanced Self-Organization Maps (EASOM), to minimize uncertainties associated with state of charge (SOC) estimation and measurements. EASOM employs a strategic approach to identify the most suitable candidate by utilizing an objective function and incorporates a fading memory forgetting factor to improve battery capacity estimation. Validation through six tests conducted in the context of hybrid electric vehicles demonstrates EASOM’s outstanding performance, with a maximum error rate of only 1.25% and consistently low predictive performance metrics below 1%. The article [34] introduces a semi-supervised learning (SSL) approach that leverages abundant unlabeled charging data, which is often overlooked. The SSL method uses two regressors to map health indicators (HIs) to SOH, augmenting training samples by predicting pseudo-labels for unlabeled data. The results demonstrate remarkable improvement in SOH estimation accuracy, with an average root-mean-square error (RMSE) of only 0.55% for seven cells using labeled data from just one cell.

### 1.1. Objective of this research

In the realm of State of Health (SOH) estimation for lithium batteries, our research distinguishes itself from the studies discussed above in several ways. While many previous approaches predominantly focus on SOC estimation or utilize specific SOC-dependent algorithms, our primary objective is to enhance SOH estimation precision independently of any particular SOC estimation method. This independence is crucial, as certain SOC estimation methods can introduce circular dependencies between SOC and SOH, potentially leading to instability.

Coulomb counting, for instance, requires an accurate estimation of total capacity, which depends on the SOH, to estimate the SOC accurately. Therefore, using Coulomb counting with the proposed approach would result in unstable and circular dependencies. However, SOC estimation techniques that rely solely on voltage measurements are suitable. Our preferred approach is to employ methods based on the Kalman filter, especially the sigma-point Kalman filters (SPKF), which can optimally combine voltage and current data and are less sensitive to errors in the SOH estimate, resulting in accurate SOC estimates. Furthermore, we can use the nominal cell capacity as a static constant within the SPKF while calculating SOC estimates and yet achieve reliable results. Relevant sources for available SOC estimation approaches can be found in [35–38].

To achieve this, we introduce a new approach for evaluating the SOH of lithium batteries, which employs a gradient-based optimizer (GBO). Our method uses GBO to minimize a specific cost function and select the best candidate for updating battery SOH with a memory fading forgetting factor. To evaluate the effectiveness of our approach, we test it in both hybrid electric vehicle (HEV) and electric vehicle (EV) applications. GBO does not impose constraints on the battery current and considers the noise present in SOC estimates. The algorithm utilizes the SOC and current data to compute an unbiased estimate of the SOH, effectively mitigating the impact of estimation and measurement noises.

With the early emergence of GBO and a thorough review of existing literature, our implementation of GBO represents the first known instance of its application for evaluating battery SOH. This innovative use of GBO highlights its potential as a powerful tool for accurately estimating battery degradation and optimizing battery performance.

The article’s main contributions could be resumed in the points below:

- A novel recursive battery SOH estimator using the GBO approach is reported. For the evaluation, simulations of a pack of batteries in HEV and EV and applications were employed. The findings have demonstrated the robustness of the approach. Based on our literature review, we can confirm that this is the first attempt to estimate battery SOH employing GBO.
- The GBO-based approach takes into consideration errors; this includes estimation noises in SOC and measurement noises as well.
- GBO is compared against robust technics (PSO-LSSV (particle swarm optimization-least square support vector regression), BCRLS-MWDEKF (BCRLS-multiple weighted dual extended Kalman filtering), TLS (Total least square), AWTLS (approximate weighted total least squares)).

## 2. Problem definition and GBO algorithm

### 2.1. Problem definition

The SOH is expressed as:

$$SOH=\frac{Q}{Q_{n}}=K*Q$$ (2)

With Q as the actual capacity and Qn is the initial capacity defined by the manufacturer (K = 1/Qn).

In the other hand, the coulomb counting is expressed as:

$$SOC(t_{1})=\frac{1}{Q}\int_{t0}^{t1}\eta i(t)dt+SOC(t_{0})$$ (3)

In the context of battery technology, η is commonly referred to as the coulombic counting coefficient, while SOC represents the state of charge and Q refers to the capacity of the battery. Additionally, i(t) is used to denote the battery current.

The linear equation Eq (4) appears when we rearrange Eq (3) and replace the capacity with SOH. Assuming we have both xj (ΔSOC) and yj (the accumulated amp hours) values, the SOH could be evaluated through a straightforward linear regression approach. The main complication is that yj and xj will always carry some noise. As a result, Eq (4) turns out to be: (yj -Δyj) = SOH (xj -Δxj).


$$\underset{y_{j}}{\underset{︸}{K\times \int_{t0}^{t1}\eta i(t)dt}}=\mathrm{SOH}\times \underset{x_{j}}{\underset{︸}{\left(SOC(t_{1})‐SOC(t_{0}\right)}}$$ (4)

In this paper, we implement the GOB algorithm to improve battery SOH. For this purpose, we need a specific cost function. We have adopted the cost function Eq (5), inspired by the one developed by G.Plett in [30]. We have used Eq (5) as a cost function, which allows us first to derive the most appropriate candidate for estimating the state of health, denoted SOHhat. Then, the SOHhat value is used via a memory forgetting factor to evaluate the SOH of the battery.


$$f_{loss}=\frac{{\left(y_{j}-SOH_{\mathrm{hat}}x_{j}\right)}^{2}}{{\left(SOH_{\mathrm{hat}}\right)}^{2}\sigma^{2}{}_{xj}+\sigma^{2}{}_{yj}}$$ (5)

*SOH*_*hat*_ is the estimated SOH. *x*_*j*_ refers to the estimated ΔSOC over [t0, t1], the *y*_*j*_ is the stored ampere hours across the same interval [t0, t1]. (σ*x*_*j*_)2 and (σ*x*_*j*_)2 are the variances on *x*_*j*_ and *y*_*j*_ axes respectively. The loss function is constrained by:

$$SOH_{low}<SOH_{hat}<SOH_{high}$$ (6)

*SOH*_*low*_ and *SOH*_*high*_ are the min and max values of the SOH.

In this paper, we utilize the gradient-based optimizer (GBO) to estimate battery SOH by minimizing the loss function Eq (5). GBO is a high-performance algorithm known for its good exploration and convergence rate. It relies on two main operators: the gradient search rule (GSR) and the local escaping operator (LEO), which scan the search area ([*SOH*_*low*_ *SOH*_*high*_]). The GSR utilizes a gradient-based approach to improve search exploration and increase the rate of convergence to find the best positions in the search area. LEO allows GBO to break out of local optima. From the best-selected solutions, the optimal candidate (*SOH*_*hat*_) is chosen and used in evaluating the SOH via a forgetting factor. Furthermore, we will present a detailed explanation of the Gradient-based optimizer (GBO).

### 2.2. Gradient-based optimizer

The GBO is a search algorithm inspired by Newton’s gradient-based method [39]. This algorithm consists of two main operators: the gradient-based search rule (GSR) and the local escape operator (LEO), as well as a collection of vectors to scan the searching space. GSR employs the gradient approach to enhance the search pattern and accelerate the convergence rate to reach a better solution within the search area. GSR is described as:

$$GSR=rand.\varepsilon_{1}(\frac{2.\Delta x.x_{n}}{x_{wr}-x_{b}+\nu })$$ (7)

*ν* being a very small number within [0, 0.1]. *x*_*b*_ and *x*_*wr*_ represent the optimal and worst solutions, *ε*_*1*_ stands for a balance coefficient expressed as:

$$\alpha =|\beta .\sin(\frac{3\pi }{2}+\sin(\beta .\frac{3\pi }{2}))|$$ (8)

$$\beta ={\left(1-{\left(\frac{m}{M}\right)}^{3}\right)}^{2}\times (\beta_{\max}-\beta_{\min})+\beta_{\min}$$ (9)

with *b*_*max*_ and *b*_*min*_ as constants equal to 1.2 and 0.2, respectively, *m* being the actual iteration and *M* the number of total iterations. To improve the utilization of the area near *x*_*n*_, the direction of motion (*DMV*) is added as well, the *DMV* is defined as follows:

$$DMV=rand.\varepsilon_{2}.(x_{b}-x_{n})$$ (10)

$$\varepsilon_{2}=2.rand.\alpha -\alpha$$ (11)

On this basis, the new agent location is formulated as:

$$x_{n+1}=DMV-GSR+x_{n}$$ (12)

GBO employs LEO to skip local optima. The positions generated at the GBO are utilized in this stage; the pseudocode for the operation is shown below in Table 1.

**Table 1:** LEO pseudocode.

*if (pr>rand)* *if (rand<0*.*5)* $X_{LEO}^{m}=f_{1}(u_{1}.xb-u_{2}.x_{k}^{m})+f_{2}.\varepsilon_{1}(u_{3}.(X2_{n}^{m}-X1_{n}^{m})-u_{2}.(x_{r1}^{m}-x_{r2}^{m}))/2+X_{n}^{m+1}$ $X_{n}^{m+1}=X_{LEO}^{m}$ else $X_{LEO}^{m}=f_{1}(u_{1}.xb-u_{2}.x_{k}^{m})-f_{2}.\varepsilon_{1}(u_{3}.(X2_{n}^{m}-X1_{n}^{m})-u_{2}.(x_{r1}^{m}-x_{r2}^{m}))/2+X_{n}^{m+1}$ $X_{n}^{m+1}=X_{LEO}^{m}$ *end* *end*

Here, pr refers to the probability, ${X2}_{n}^{m}$ and ${X1}_{n}^{m}$ are solutions produced by GBO for m candidate and n optimizing variables, $X_{r2}^{m}$ and $X_{r1}^{m}$ are two solutions chosen randomly, f1 is a uniform number chosen randomly between 1 and -1, f2 is a uniform random number derived from a normal distribution with a zero mean and standard deviation 1. The rest of the parameters are set as:

$$u_{1}=\{\begin{array}{l} 2\times rand\;if\;\mu_{1}<0.1\\ otherwise\;u_{1}=1 \end{array}$$ (13)

$$u_{2},u_{3}=\{\begin{array}{l} rand\;if\;\mu_{1}<0.1\\ otherwise\;1 \end{array}$$ (14)

**Fig 1:**
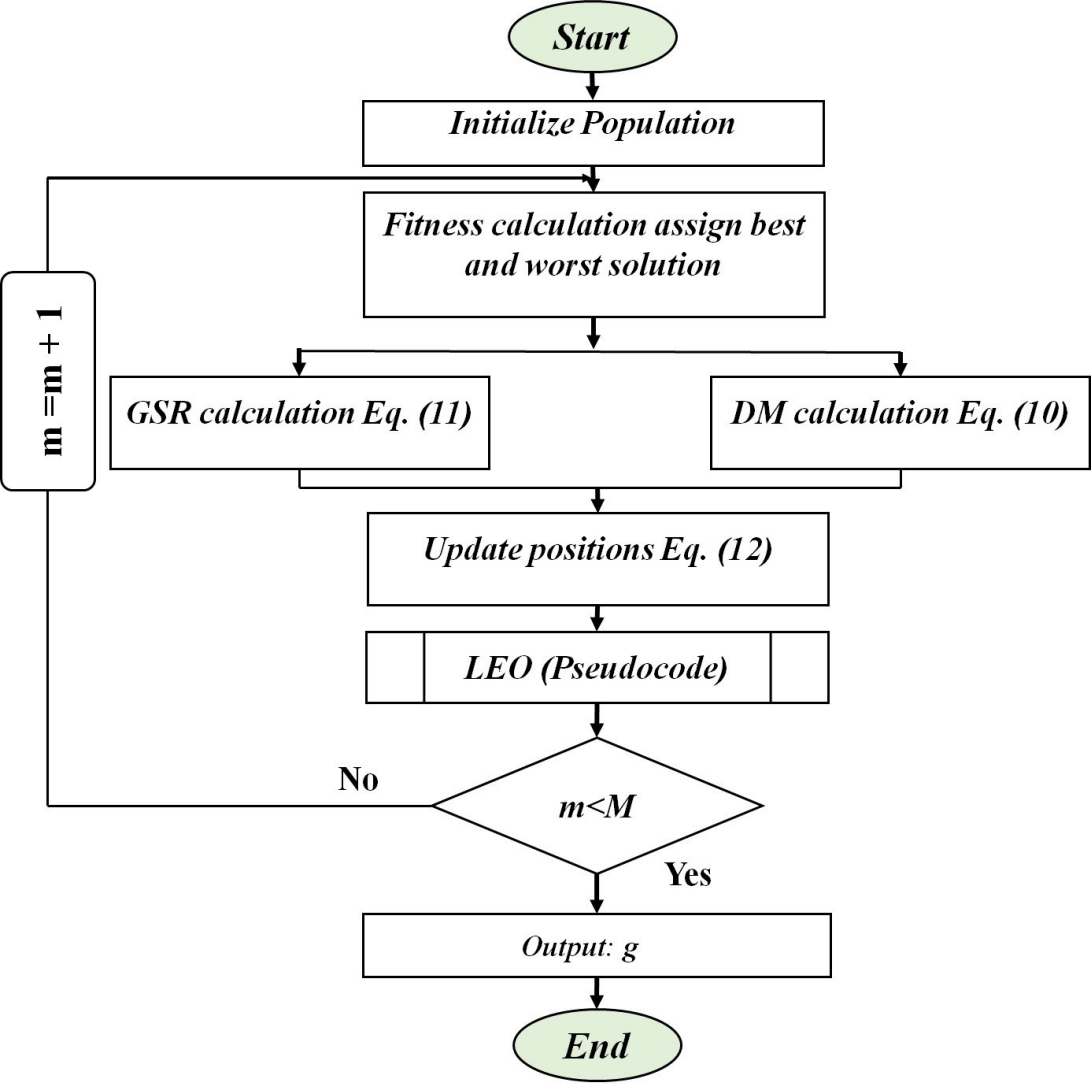
GBO procedure.

*u*_*1*_, *u*_*2*_, and *u*_*3*_ represent a random number between 0 and 1. The algorithm procedure is depicted in Fig 1.

### 2.3 The proposed framework

**Fig 2:**
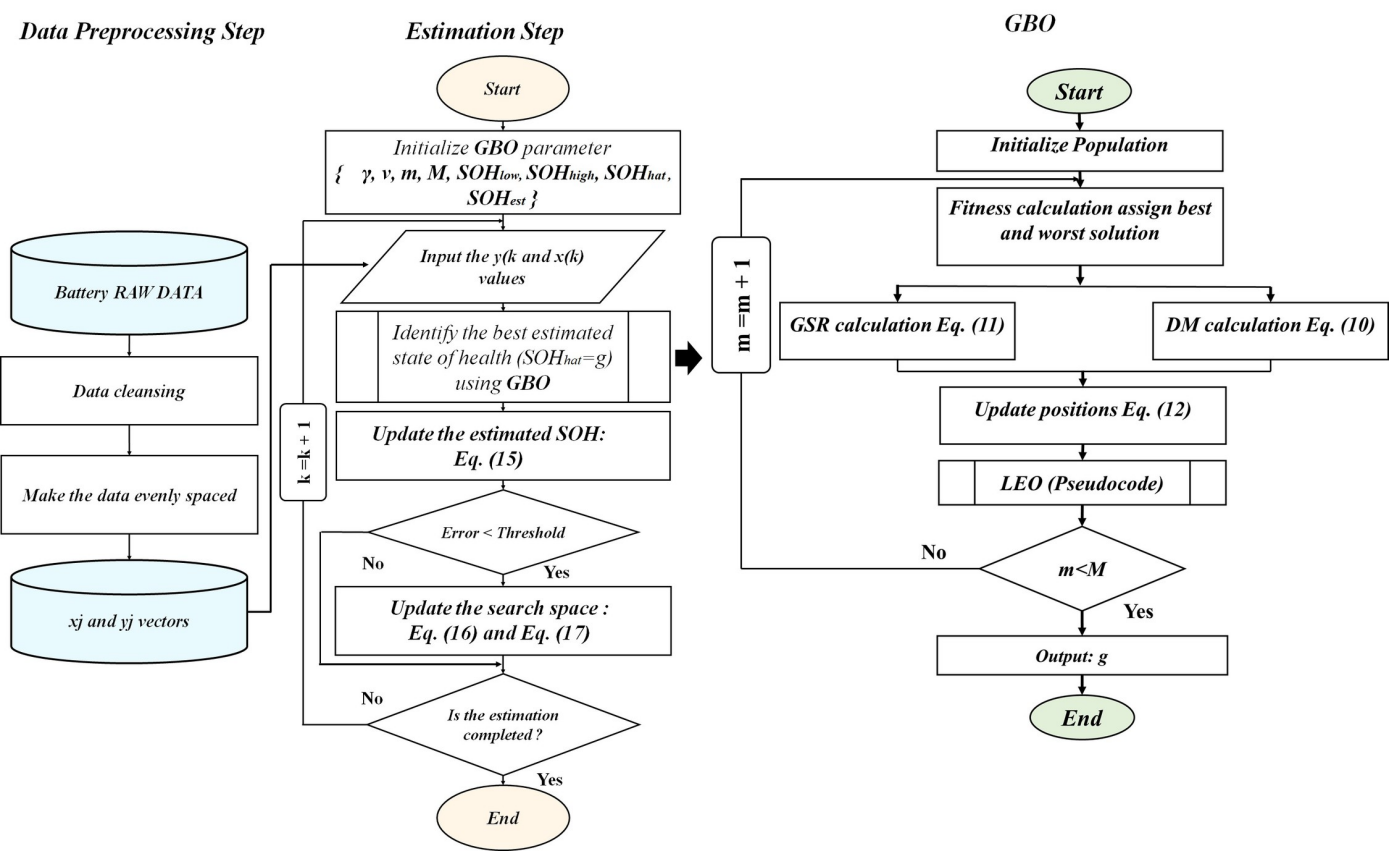
GBO framework used to estimate the battery SOH.

A sketch of the overall framework can be viewed in Fig 2. The whole approach is split into two parts: pre-treatment and estimation.

Phase 1: In any data analysis process, the reliability and accuracy of the dataset are paramount. Therefore, clearing unreliable records is a critical phase involving cleaning the data to ensure its readiness for treatment. The process entails eliminating all potentially incorrect, overlapping, or erroneous entries from the dataset to guarantee its quality. The estimation of SOC is not the main focus of this study, and it is approached using a separate methodology to avoid circular dependencies with SOC. In this research, SOC estimation techniques relying solely on voltage measurements are employed, specifically the sigma-point Kalman filters (SPKF). By effectively incorporating current and voltage data, SPKF minimizes errors in capacity estimation and enables accurate SOC estimation. Additionally, other techniques for SOC prediction, as outlined in [35–38], can also be explored. Afterward, the net resultant data are evenly distributed to ensure unbiased results before calculating the *x*_*j*_ and *y*_*j*_ vectors.

Phase 2: The GBO is deployed to assess the SOH. Initially, the algorithm begins by defining the parameters of GBO. With each subsequent *x*_*j*_ and *y*_*j*_ value, GBO proceeds to locate the highest candidate (*SOH*_*hat*_) in an effort to keep Eq (5) (the loss function) to a minimum. We embedded a forgetting factor γ in Eq (15) (typically ranging from 0.9 to 1 [40–44]) to compute the *SOH*_*est*_ (estimated health state), γ makes sure that GBO converges to the true value rapidly.


$${\mathrm{SOH}}_{\mathrm{est}}(k)=(1-\gamma ){.\mathrm{SOH}}_{hat}+\gamma .{\mathrm{SOH}}_{\mathrm{est}}(k-1)$$ (15)

To control the search space effectively, we have implemented an additional step in our methodology. This step involves modifying the parameter boundaries *SOH*_*low*_ and *SOH*_*high*_ whenever the error exceeds a predetermined threshold. To accomplish this, we utilize two equations to adjust the parameter boundaries as necessary:

$$SOH_{low}=SOH_{low}+\chi (SOH_{high}-SOH_{low})$$ (16)

$$SOH_{high}=SOH_{high}-\chi (SOH_{high}-SOH_{low})$$ (17)

## 3. Hybrid and electric vehicle tests

We used a selection of different scenarios elaborated by Plett [29,30] to assess GBO HEV and EV tests), we also compare GBO against (PSO-LSSV [45], BCRLS-MWDEK [46], TLS and AWTLS [29,30]) in term of SOH estimation performance.

Under the first set of simulations, scenarios of hybrid electric vehicles are considered. From a SOH estimation perspective, these applications are generally characterized by a narrow SOC window. For the following tests, it is assumed that the battery has a capacity of 100 Ah. Table 2 displays the values of GBO parameters for the HEV case. The xj (ΔSOC) and yj (∫ i: the accumulated current) are produced as discussed below.

**Table 2:** GBO parameters for HEV.

Scenario	Population number (N) ^a^	number of Decision variables ^b^	Number of iterations Maxgen ^c^	Forgetting factor γ ^d^	Beta β ^e^	Threshold α ^f^	SOH (%) ^g^	
							SOH_low_ (%) ^h^	SOH_high_ (%) ^i^
**#HEV1**	10	1	10	0.9990	0.0002	0.0070	1	100
**#HEV2**	10	1	10	0.9990	0.0002	0.0070	26	100
**#HEV3**	10	1	10	0.9980	0.0002	0.005	3	100
**#HEV4**	10	1	10	0.9969	0.0002	0.001	9	100
**#HEV5**	10	1	10	0.9960	0.00026	1.e-07	0	100

^a^ Population number (N): Represents the size of the population, indicating the number of individual solutions in each generation.

^b^ Number of Decision Variables: Indicates the count of variables or parameters being optimized by the GBO algorithm.

^c^ Maxgen: Specifies the maximum number of iterations of GBO.

^d^ Forgetting factor (λ): Represents the memory fading or forgetting rate.

^e^ Beta threshold (β): Refers to a threshold value used in the GBO algorithm to control the exploration and exploitation balance.

^f^ Alpha threshold (α): Denotes a threshold value utilized in the GBO algorithm to determine the selection of the optimal candidate for updating the battery’s SOH.

^g^ SOH (%): Represents the SOH percentage.

^h^ SOH low (%): Specifies the lower limit of the SOH.

^i^ SOH high (%): Indicates the upper limit of the SOH.

The purpose of these tests is to evaluate the performance of the algorithms in terms of the SOH prediction for hybrid electric vehicles applications. The test is focused on HEV that operate within a SOC range of 60% to 40%, which is a common range for these vehicles. During the process of updating the SOH, changes in SOC can vary within +0.2 or -0.2. This means that the battery’s charge level can fluctuate by up to 20% during the test [29,30].

**Fig 3:**
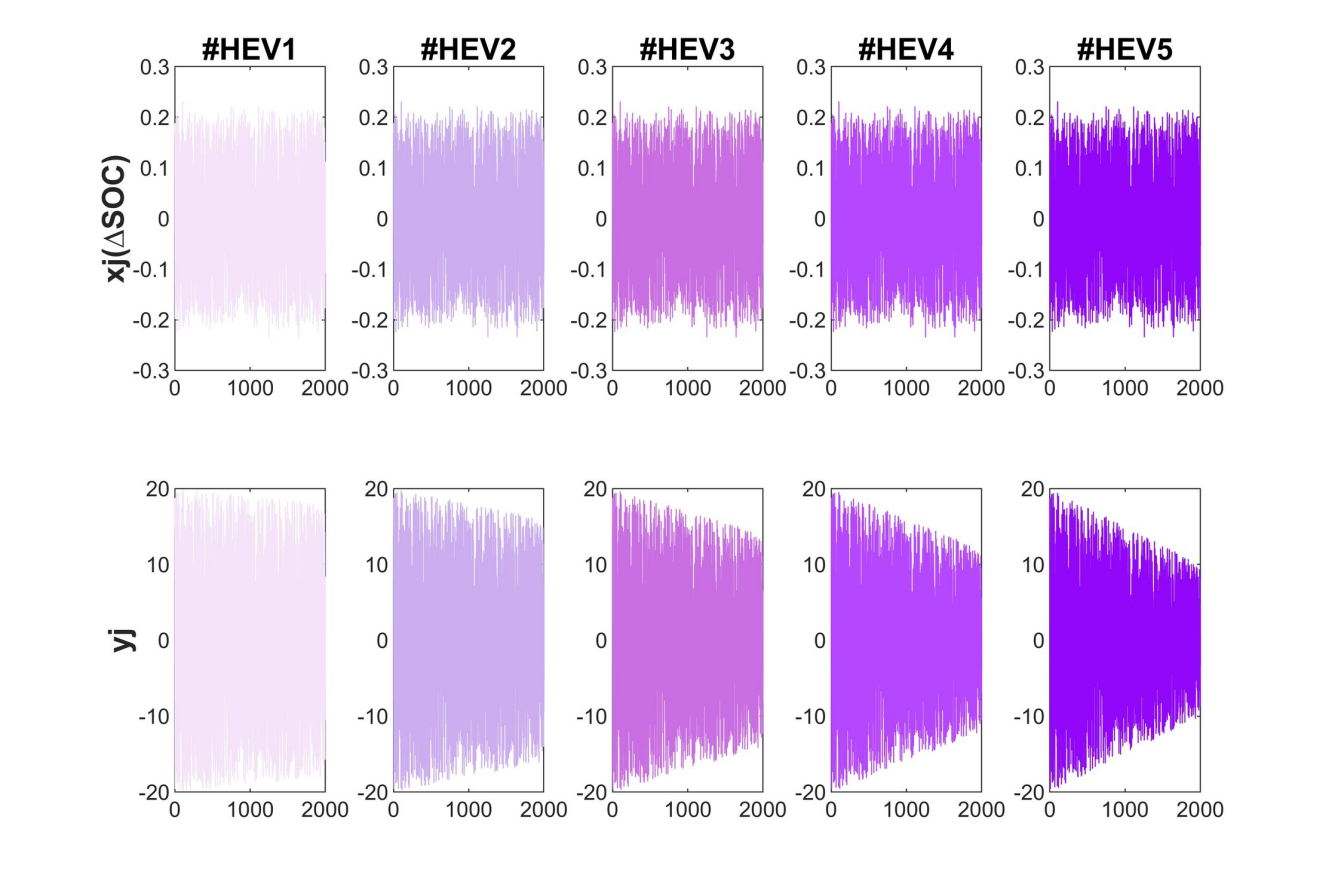
*x*_*j*_, and *y*_*j*_ variables for HEV tests.

The primary objective of the test is to assess the effectiveness of algorithms in monitoring the changes in SOH. SOH is an important parameter for electric vehicles, as it determines the battery’s capacity and overall health. The changes in SOH during the test follow a slope of -0.005/Qn, -0.01/Qn, -0.015/Qn, -0.02/Qn, and -0.025/Qn, corresponding to a decrease in SOH by 1.5%, 3%, 4.5%, 6%, and 7.5% (every 300 iterations as depicted in Fig 3), respectively. This is considered quite an aggressive slope, as it represents a significant decrease in the battery’s SOH over a short period [29,30].

## 4. Electric vehicle tests

In this section, we will examine the remaining experiments, which are designed to simulate batteries for PHEV and EV operations. These scenarios are more challenging, as they involve larger cell capacities and higher SOC values. In all cases, we assume a battery with a capacity (Q) of 300 Ah, a maximum rate of ±5Qnom, and a 10-bit resolution current sensor [29,30]. GBO parameters for the EV case are given in Table 3.

**Table 3:** GBO parameters for HEV.

Scenario	Population number (N)	number of Decision variables	Number of iterations Maxgen	Forgetting factor γ	beta	Threshold α	SOH (%)	
							SOH_low_ (%)	SOH_high_ (%)
**#EV1**	10	1	10	0.9977	0.0002	0.02	52	100
**#EV2**	10	1	10	0.9970	0.0002	0.0096	0	100
**#EV3**	10	1	10	0.9946	0.0002	0.0089	38	100
**#EV4**	10	1	10	0.9922	0.0002	0.007	44	100
**#EV5**	10	1	10	0.9870	0.0002	0.006	34	100

**Fig 4:**
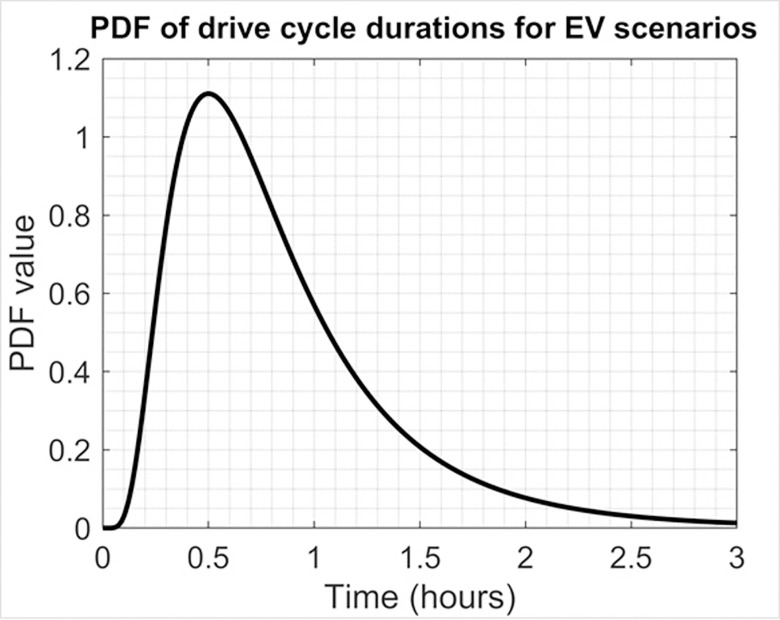
#EV PDF (drive cycle durations).

The experiments involve continuously updating the battery’s SOH during vehicle operation. We express the variable mi as a lognormal random quantity, which generates random updates for the *x*_*j*_ and *y*_*j*_ variables. This approach produces a probability density function (as shown in Fig 4) that is designed to provide satisfactory drive durations across a range of driving conditions and behaviors.

**Fig 5:**
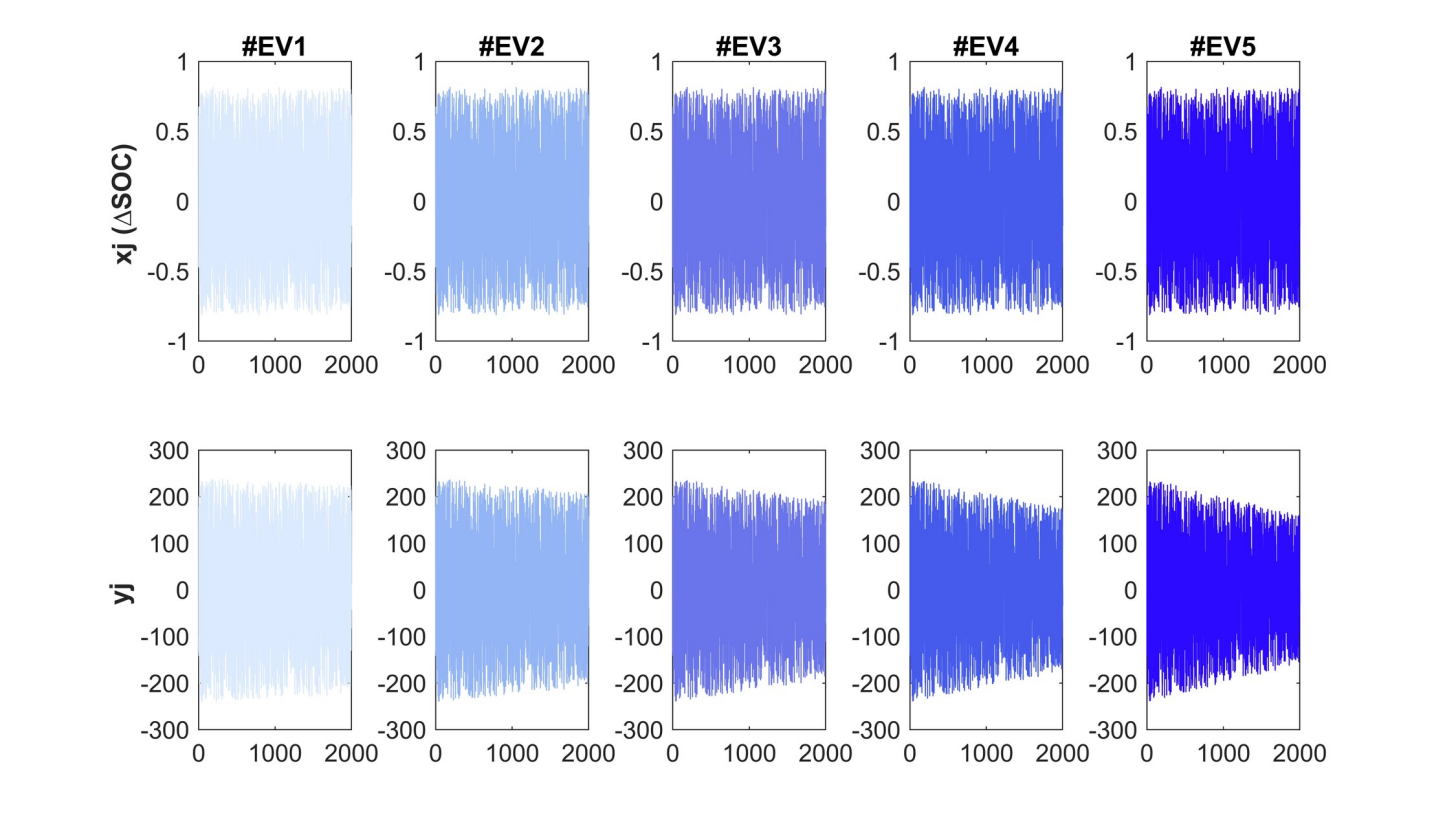
*x*_*j*_(ΔSOC), and *y*_*j*_(accumulated current) variables for HEV tests.

The driving cycle in these experiments utilizes a significant portion of the battery pack, which requires an update interval that is not periodic. To achieve this, we establish a SOC interval of ±80%, with *x*_*j*_ (ΔSOC) being a variable that randomly varies between -0.8 and +0.8 (as depicted in Fig 5). The curve of the SOH has a gradient of -0.01/Qn, -0.02/Qn, -0.03/Qn, -0.04/Qn, and -0.05/Qn (where Qn = 300 Ah), which means that the SOH is declining by 1%, 2%, 3%, 4%, and 5% (every 300 iterations), respectively [29,30].

## 5. Results and discussion

**Fig 6:**
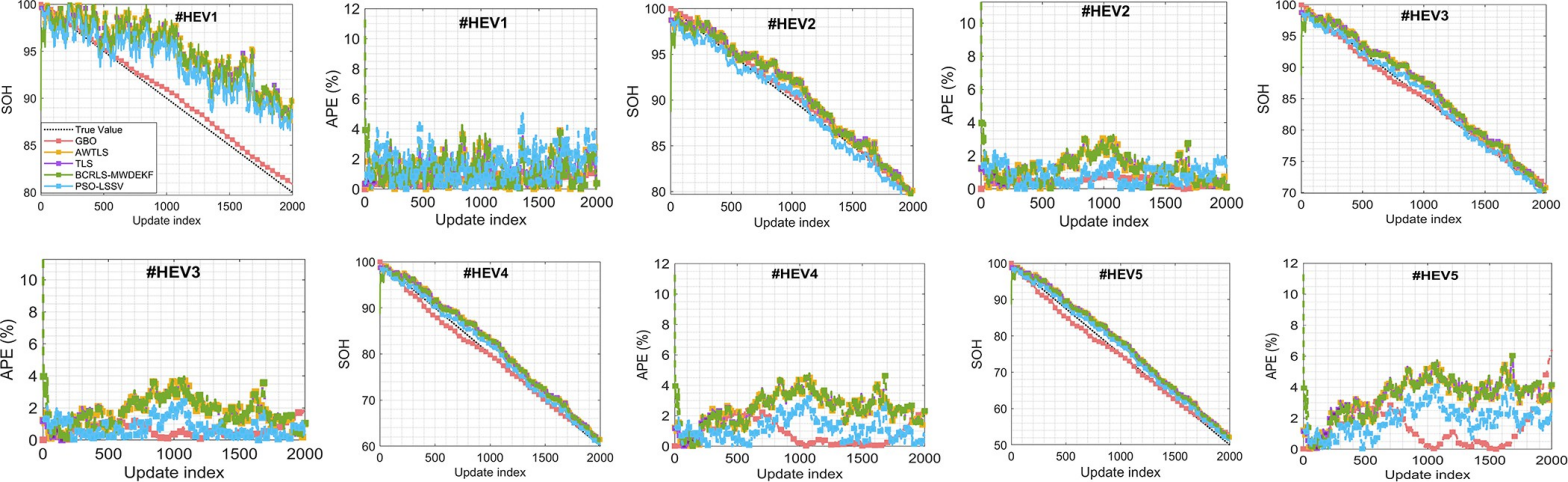
SOH estimation and APE in percent. (a, b) # HEV1, (c, d) # HEV2, (e, f) # HEV3, (g, h) # HEV4, (i, j) # HEV5.

**Fig 7:**
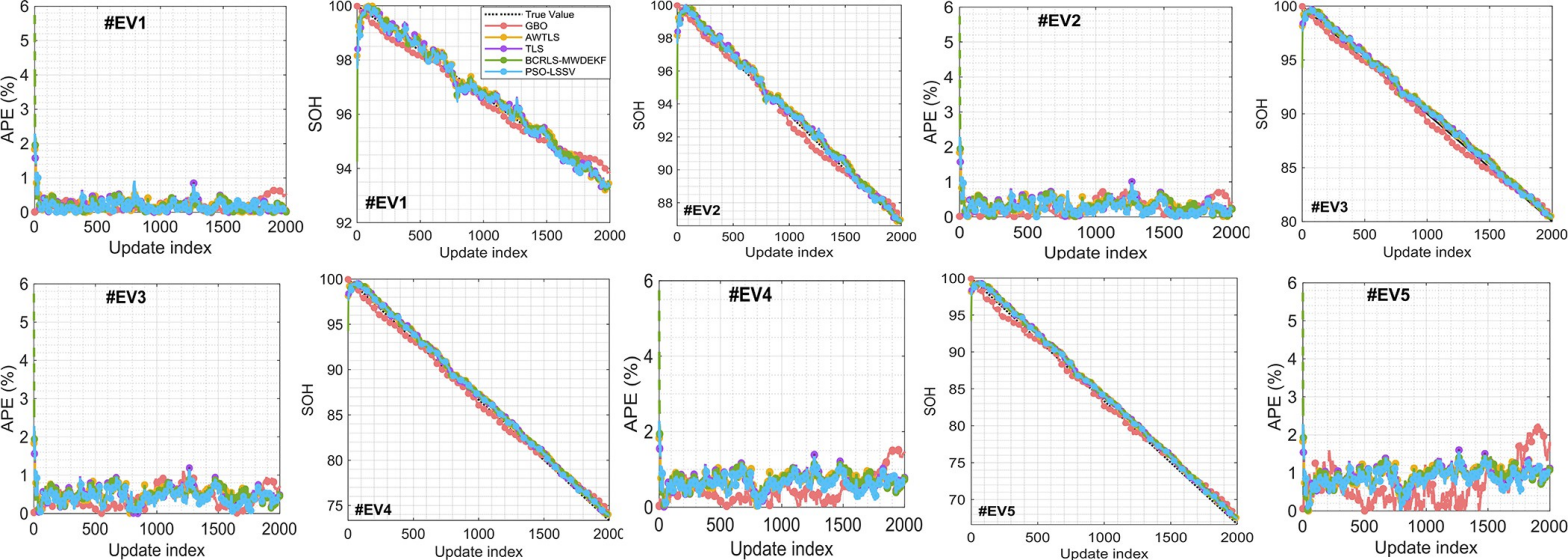
SOH estimation and APE in percent. (a, b) # EV1, (c, d) # EV2, (e, f) # EV3, (g, h) # EV4, (i, j) # EV5.


To comprehensively assess the performance of the different algorithms in estimating the State of Health (SOH) of batteries in both Hybrid Electric Vehicle (HEV) and Electric Vehicle (EV) scenarios, we conducted an in-depth analysis of the Absolute Percentage Error (APE) expressed in percentage. These findings are graphically presented in Figs 6 and 7 alongside the SOH estimates generated by all the algorithms.

$$\mathrm{APE}=|\frac{{\mathrm{SOH}}_{true}(j)-{\mathrm{SOH}}_{est}(j)}{{\mathrm{SOH}}_{true}(j)}|*100$$ (18)

Upon meticulous examination, it becomes evident that the Gradient-Based Optimizer (GBO) algorithm consistently exhibits a lower APE (as defined in Eq (18)) when compared to the other methods in both HEV and EV scenarios (Figs 6 and 7). This reduction in error is particularly noteworthy, given the challenging nature of the test scenarios.

One of the primary challenges in these scenarios is the wide and aggressive variation in the State of Charge (SOC) level. In HEV scenarios, SOC fluctuates erratically within a range of ±40%, while in EV scenarios, it experiences even more substantial fluctuations, ranging from ±80%. This erratic SOC behavior significantly contributes to the high errors observed across all methods (Figs 6B, 6D, 6F, 6H, 6J, 7B, 7D, 7F, 7H and 7J), as outlined in Table 4. Moreover, these fluctuations impose substantial obstacles on the convergence rate of all the algorithms.

**Table 4:** MEAN and MAX error in (%).

Scenarios	Performance indicators	GBO	AWTLS	TLS	BCRLS-MWDEKF	PSO-LSSV
**#HEV1**	Max (%)	1.11	11.48	11.48	11.48	9.8
	Mean (%)	0. 61	5.26	5.25	5.32	4.1
**#HEV2**	Max (%)	0.81	2.33	2.33	11.27	2.62
	Mean (%)	0.39	1.05	1.05	1.11	0.75
**#HEV3**	Max (%)	1.34	3.81	3.8	11.27	2.27
	Mean (%)	0.49	1.46	1.45	1.51	0.63
**#HEV4**	Max (%)	2.17	5.18	5.18	11.26	3.68
	Mean (%)	0.71	1.92	1.92	1.95	0.86
**#HEV5**	Max (%)	3.21	6.13	6.12	11.26	4.67
	Mean (%)	1.03	2.41	2.40	2.42	1.33
**#EV1**	Max (%)	0.65	2.08	2.26	5.73	2.28
	Mean (%)	0. 21	0. 19	0.20	0.19	0.19
**#EV2**	Max (%)	0.74	2.07	2.25	5.73	2.28
	Mean (%)	0.24	0.31	0.29	0. 31	0.26
**#EV3**	Max (%)	0. 97	2.05	2.24	5.72	2.26
	Mean (%)	0.35	0.46	0.44	0.46	0.40
**#EV4**	Max (%)	1.16	2.03	2.22	5.71	2.24
	Mean (%)	0.40	0.61	0.60	0.62	0.55
**#EV5**	Max (%)	1.57	2.13	2.18	5.69	2.20
	Mean (%)	0. 57	0. 77	0. 76	0. 78	0.71

In both HEV and EV scenarios, the battery’s SOH undergoes a steep decline following a gradient ranging from -0.005/Qn to -0.025/Qn for HEV and -0.01/Qn to -0.05/Qn for EV. This aggressive and rapid SOH degradation is both unusual and challenging.

Despite the formidable challenges posed by the unpredictable and aggressive nature of the tests, the GBO algorithm consistently outperforms other methods. It manages to maintain an overall error rate that is lower than that of its counterparts. This exceptional performance underscores the GBO algorithm’s capacity to deliver precise predictions even in complex, uncertain, and challenging environments.

Our detailed analysis, as presented in Table 4, reveals further insights into the algorithms’ performance in HEV scenarios. GBO emerges as the top-performing method, with maximum errors ranging between 0.82% and 3.21%. The second-best method, PSO-LSSV, records maximum errors within the range of 2.27% to 9.8%. Conversely, AWTLS, TLS, and BCRLS-MWDEKF consistently exhibit the highest maximum errors across all scenarios.

In terms of mean errors in HEV scenarios, both GBO and PSO-LSSV consistently outperform other algorithms, maintaining lower mean errors. AWTLS, TLS, and BCRLS-MWDEKF exhibit mean errors that are generally higher than those of GBO and PSO-LSSV across all scenarios.

Similarly, in EV scenarios, GBO stands out with the lowest maximum errors, ranging from 0.65% to 1.57%. PSO-LSSV follows closely with maximum errors ranging from 2.20% to 2.28%. AWTLS, TLS, and BCRLS-MWDEKF consistently show higher maximum errors compared to GBO and PSO-LSSV.

The data presented in Table 4 underscores GBO’s ability to accurately capture the intricate properties of online SOH changes during battery operation. In comparison, GBO surpasses the other techniques, demonstrating its remarkable potential for precise SOH predictions across various scenarios.

In conclusion, the results validate the superiority of the GBO algorithm over the other methods in accurately predicting battery SOH in both HEV and EV scenarios, even in the face of challenging and erratic operating conditions. These findings further solidify GBO’s standing as a reliable tool with extensive applications, particularly in the realms of Hybrid Electric Vehicles (HEVs) and Electric Vehicles (EVs), where precise SOH estimation is crucial. Additionally, GBO’s versatility, with its compatibility across multiple programming languages, positions it as an ideal choice for a wide array of applications.

To evaluate the efficacy of the algorithms, we assess their predictive capability using three variables: Mean Squared Error (MSE), Mean Absolute Percentage Error (MAPE), and Root Mean Squared Error (RMSE). These allow for a comprehensive assessment of the algorithms’ performance, providing valuable insights into how accurately they predict battery SOH:

$$\mathrm{MSE}=\frac{1}{n}{\sum_{j=1}^{n}\left(SOH_{true}(j)-SOH_{est}(j)\right)}^{2}$$ (19)

$$\mathrm{MAPE}\;(\%)=\frac{100}{n}\sum_{j=1}^{n}\frac{\left|SOH_{true}(j)-SOH_{est}(j)\right|}{SOH_{true}(j)}$$ (20)

$$\mathrm{RMSE}=\sqrt{\frac{1}{n}{\sum_{j=1}^{n}\left(SOH_{true}(j)-SOH_{est}(j)\right)}^{2}}$$ (21)

n: the actual number of cycles, *SOH*_*est*_ and *SOH*_*true*_: the estimated and actual SOH of the battery.

The results presented in Table 5 demonstrate the superiority of the GBO algorithm over the TLS methods in accurately predicting the battery SOH in both Hybrid Electric Vehicle (HEV) and Electric Vehicle (EV) scenarios:

**Table 5:** Predictive performance indicators.

Scenarios	Performance indicators	GBO	AWTLS	TLS	BCRLS-MWDEKF	PSO-LSSV
**#HEV1**	RMSE (%)	0.67	5.99	5.99	6.01	4.78
	MAPE	0.6961	5.6027	5.6027	5.6640	4.4406
	MSE	4.5317e-05	0.0036	0.0036	0.0036	0.0023
**#HEV2**	RMSE (%)	0.44	1.27	1.27	1.37	0.90
	MAPE	0.43	1.15	1.15	1.21	0.84
	MSE	1.9451e-05	1.6174e-04	1.6174e-04	1.8642e-04	8.0939e-05
**#HEV3**	RMSE (%)	0.59	1.65	1.64	1.72	0.8
	MAPE	0.5957	1.7003	1.700	1.7502	0.7249
	MSE	3.5207e-05	2.7347e-04	2.7345e-04	2.9548e-04	6.4465e-05
**#HEV4**	RMSE (%)	0.97	2.009	2	2.13	1.05
	MAPE	0.86	2.39	2.39	2.43	1.07
	MSE	9.4060e-05	4.3673e-04	4.367e-04	4.5416e-04	1.0931e-04
**#HEV5**	RMSE (%)	1.35	2.56	2.55	2.58	1.47
	MAPE	1.3912	3.2471	3.247	3.2591	1.8176
	MSE	1.8130e-04	6.5482e-04	6.548e-04	6.6571e-04	2.1548e-04
**#EV1**	RMSE (%)	0.25	0.26	0.28	0.30	0.27
	MAPE	0.22	0.19	0.20	0.19	0.19
	MSE	6.4827e-06	6.5157e-06	7.5692e-06	9.1077e-06	7.2391e-06
**#EV2**	RMSE (%)	0.31	0.36	0.36	0.29	0.33
	MAPE	0.26	0.32	0.31	0.33	0.27
	MSE	9.5411e-06	1.2639e-05	1.2912e-05	1.5255e-05	1.1040e-05
**#EV3**	RMSE (%)	0.41	0.49	0.49	0.52	0.45
	MAPE	0.39	0.50	0.48	0.50	0.43
	MSE	1.7045e-05	2.4105e-05	2.3763e-05	2.6733e-05	2.0349e-05
**#EV4**	RMSE (%)	0.48	0.64	0.63	0.66	0.59
	MAPE	0.47	0.70	0.68	0.71	0.63
	MSE	2.3179e-05	4.0986e-05	4.0194e-05	4.3615e-05	3.5166e-05
**#EV5**	RMSE (%)	0.70	0.80	0.79	0.81	0.74
	MAPE	0.72	0.93	0.91	0.93	0.85
	MSE	4.8376e-05	6.3369e-05	6.2294e-05	6.5987e-05	5.5491e-05

In the HEV scenarios (#HEV1 to #HEV5), the Gradient-Based Optimizer (GBO) consistently stands out as the top-performing algorithm. It exhibits the lowest RMSE, which ranges from 0.44% to 1.35%, indicating its remarkable accuracy in estimating battery SOH. Comparatively, the other algorithms, including AWTLS, TLS, BCRLS-MWDEKF, and PSO-LSSV, show higher RMSE values, with the highest reaching 2.58%.

The MAPE values follow a similar pattern, with GBO consistently maintaining the lowest error percentages, ranging from 0.43% to 1.3912%. In contrast, the other algorithms exhibit higher MAPE values, with the highest reaching 3.2591%.

The MSE values reinforce GBO’s superior performance, consistently recording the lowest values across all HEV scenarios. This underscores GBO’s ability to provide accurate and precise estimates of battery SOH in these challenging scenarios.

In the EV scenarios (#EV1 to #EV5), GBO continues to exhibit exceptional performance. It consistently achieves the lowest RMSE values, ranging from 0.25% to 0.70%. This indicates its consistent accuracy in estimating battery SOH in EV scenarios.

The MAPE values in the EV scenarios further support GBO’s dominance, with the lowest error percentages consistently attributed to GBO, ranging from 0.19% to 0.93%. In contrast, the other algorithms exhibit higher MAPE values, with the highest reaching 0.93%.

Once again, the MSE values align with the trend observed in the RMSE and MAPE, with GBO consistently recording the lowest MSE values across all EV scenarios.

The numerical results presented in Table 5 consistently demonstrate that the GBO algorithm outperforms AWTLS, TLS, BCRLS-MWDEKF, and PSO-LSSV in accurately estimating battery SOH across a diverse range of scenarios. GBO’s ability to consistently achieve lower RMSE, MAPE, and MSE values highlights its superior predictive capabilities, even in challenging and fluctuating environments.

**Fig 8:**
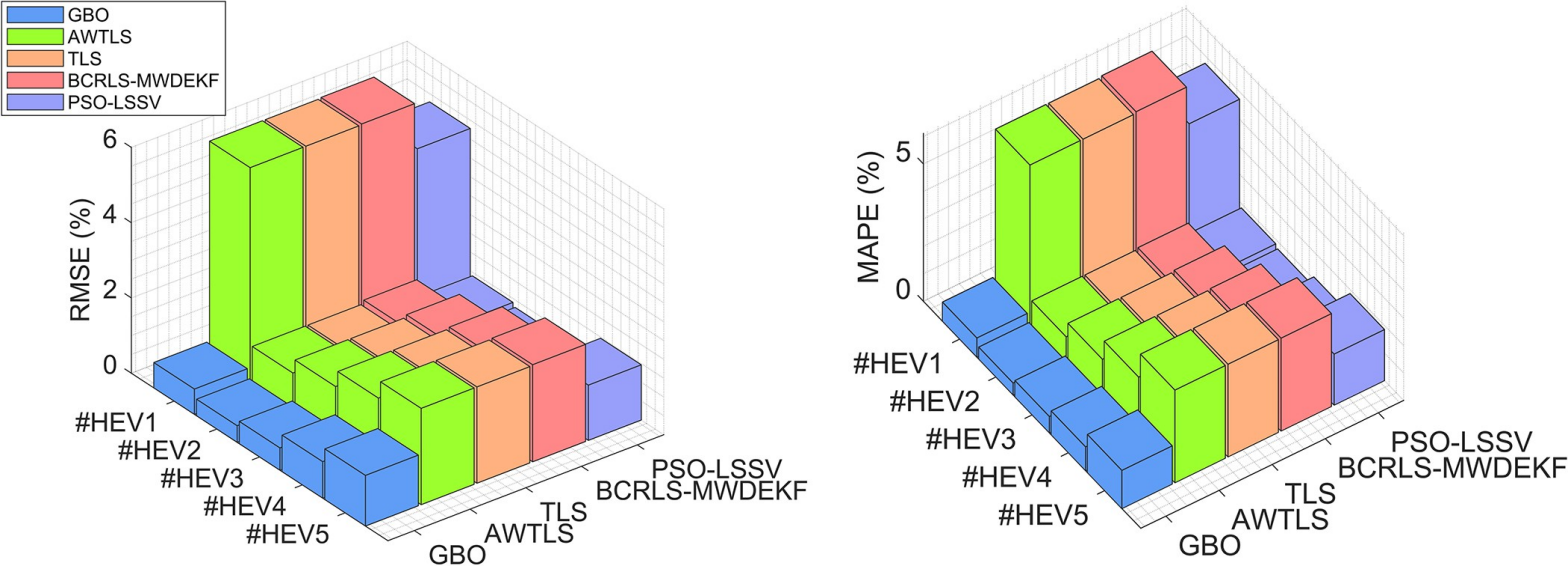
RMSE (a) and MAPE (b) for HEV scenario.

**Fig 9:**
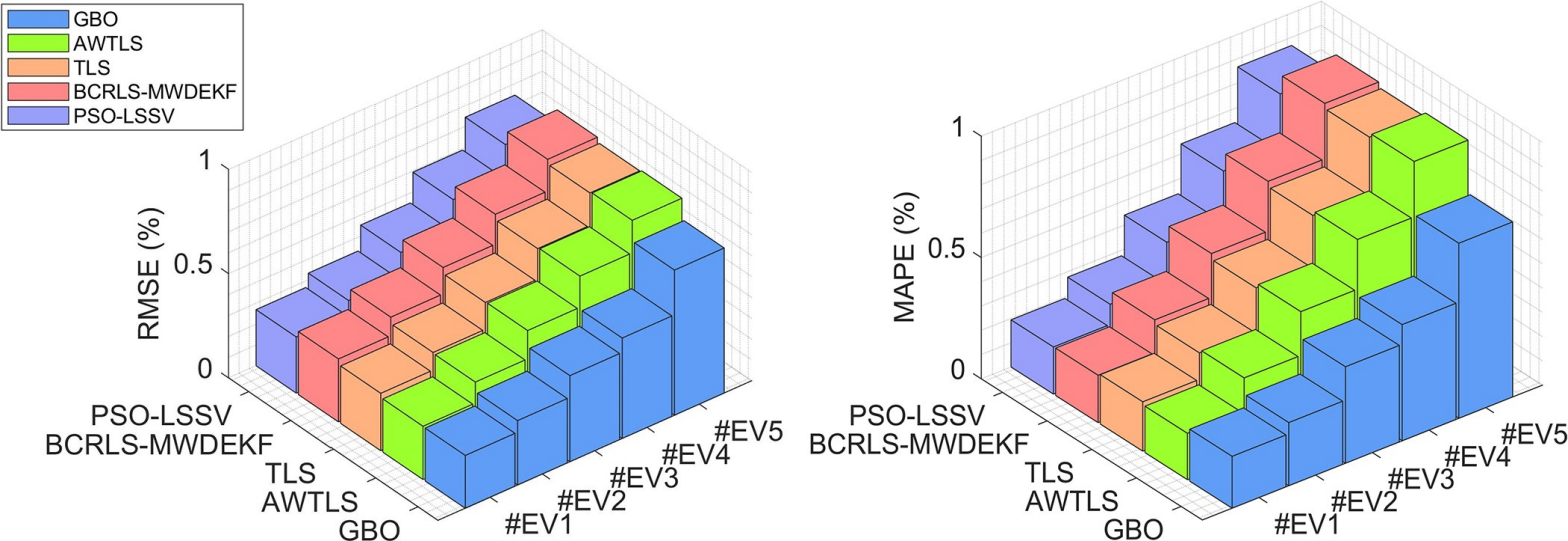
RMSE (a) and MAPE (b) for EV scenarios.

These results reinforce the conclusion that GBO is a robust and reliable algorithm for battery SOH estimation, with the potential for a wide range of applications, particularly in the context of Hybrid Electric Vehicles (HEVs) and Electric Vehicles (EVs), where precise SOH estimation is critical. GBO’s consistent accuracy and reliability position it as a valuable tool for enhancing battery health monitoring and management in real-world scenarios. Figs 8 and 9 illustrate the MAPE and RMSE values of all the methods under consideration. These figures demonstrate the remarkable accuracy of the GBO algorithm in both scenarios, with the other methods falling short in comparison.

**Fig 10:**
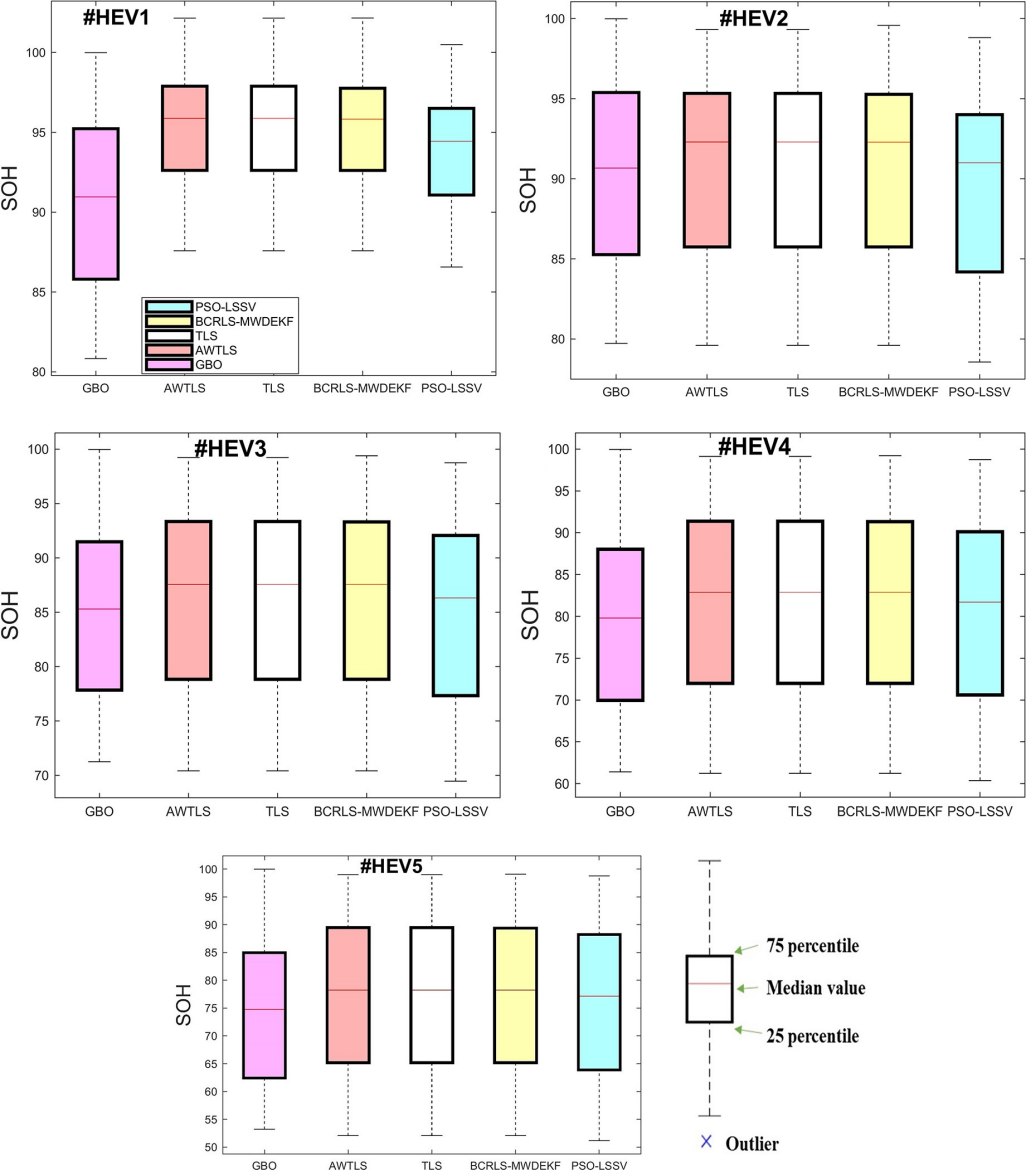
Boxplots in HEV tests. (a) #HEV1, (b) #HEV2, (c) #HEV3, (d) #HEV4, (e) #HEV5.

**Fig 11:**
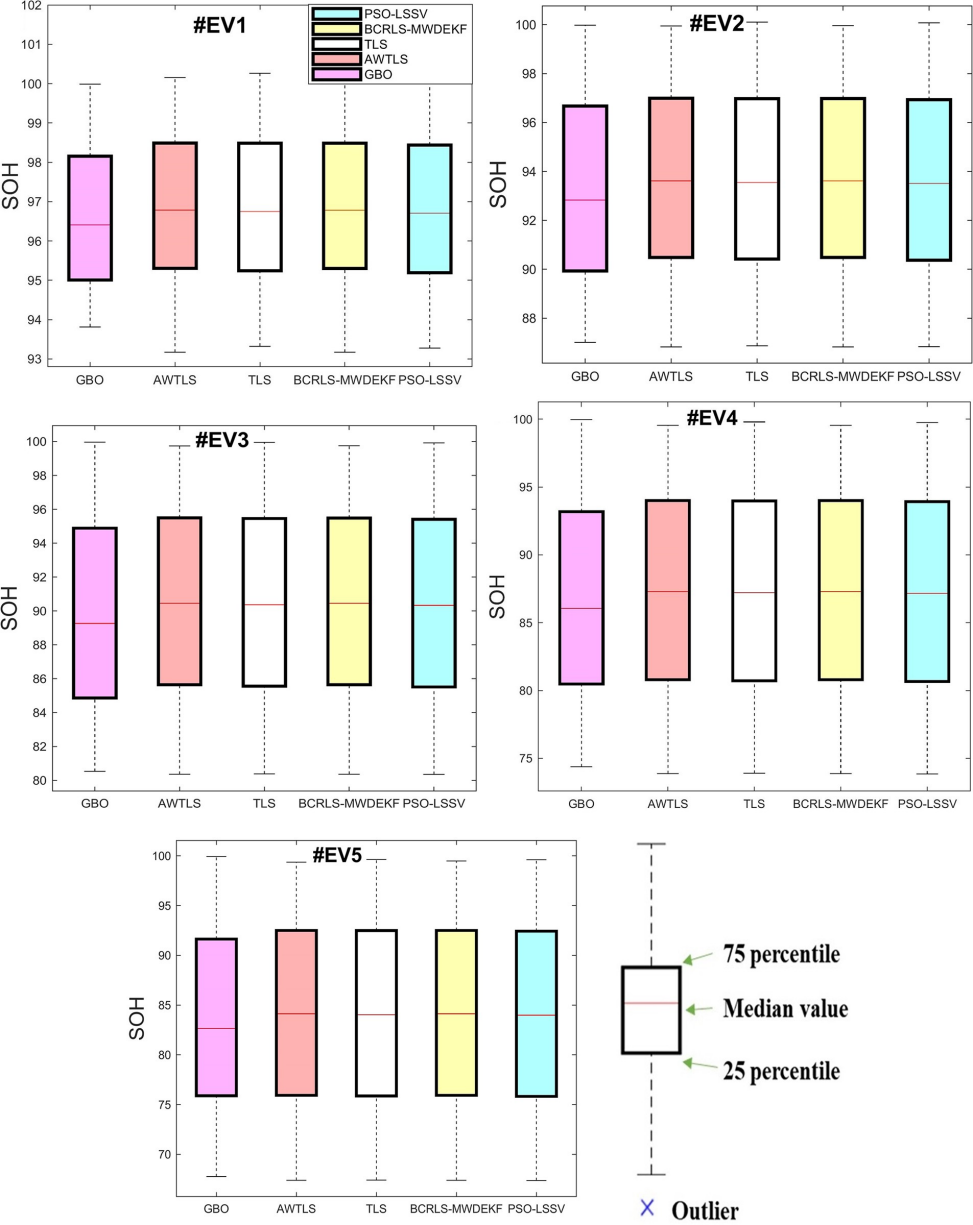
Boxplots in EV tests. (a) #EV1, (b) #EV2, (c) #EV3, (d) #EV4, (e) #EV5.

The boxplots in Figs 10 and 11 illustrate the estimated SOH of the battery during all tests for every algorithm. In each boxplot, the red horizontal line denotes the mean value of the SOH for each approach. The distance from the top edge to the bottom edge of the box is defined as the Interquartile Range (IQR). Values that lie outside the boxplot’s whiskers (the top and bottom lines) are considered outliers. The boxplots display the variability and spread of the data for each method, providing further insight into their performance and accuracy.

The GBO algorithm had the lowest spread, with the interquartile range being considerably lower than the other methods. On the contrary, the compared methods exhibited a wider spread, as evidenced by their significantly larger interquartile ranges. This suggests a greater degree of variability which could be attributed to their lower accuracy compared to the GBO algorithm.

The boxplots illustrate that all algorithms, without exception, exhibited no outliers in all tests, which indicates a higher level of accuracy in estimating the SOH. This indicates that the overall estimated SOH points of the GBO algorithm were close to the median and thus, closer to the actual true SOH.

According to the boxplots, the GBO algorithm had a relatively small difference between the 25th and 75th percentile. Tables 4 and 5 also demonstrate that GBO had small errors, with nearly 75% of the points being close to the true SOH. This indicates the precision of the algorithm, providing further evidence of its capacity for accurate updates.

As is shown in the findings above, across all tests, the GBO algorithm exhibited high performance in prediction, which could be anticipated given its working principle:

The gradient-based optimizer (GBO) algorithm is designed to efficiently navigate the search space and obtain accurate solutions using the gradient-based search rule (GSR) and local escape operator (LEO). GSR enhances the rate of convergence towards a better solution by optimizing the search pattern. In each cycle, GBO searches for the best candidate (*SOH*_*hat*_) based on the current *x*_*j*_ (ΔSOC) and *y*_*j*_ (accumulated current) values, minimizing the loss function shown in Eq (5).

GBO utilizes various parameters, such as population number (*N*), number of iterations (*Maxgen*), and forgetting factor (γ), to reach the actual value faster and with less effort. Dynamically varying the search space [*SOH*_*low*_ *SOH*_*high*_] is another factor that aids in achieving this goal. GBO’s efficient and reliable operating principle has undergone extensive testing and has demonstrated strong prediction performance in all scenarios.

By considering the intricate characteristics of a battery’s components, such as its capacity and voltage, the GBO algorithm has effectively tracked the *SOH* value of a battery cell during charging and discharging. Our approach has yielded highly accurate predictions.

The results of our study demonstrate that GBO is a potent tool with great potential for various applications, including both Hybrid Electric Vehicle (HEV) and Electric Vehicle (EV) scenarios. Additionally, GBO’s flexibility in being able to be implemented in multiple programming languages makes it an ideal choice for a wide range of applications.

## 6. Computation speed

In this assessment, we conducted a comparison between the calculation efficiency of the GBO algorithm and the compared algorithms. MATLAB software was utilized for the experiments, employing a laptop equipped with an i7-6700HQ CPU @ 2.60GHz and 8 GB of RAM. The execution of the algorithms allowed us to record the average runtime for a single cycle, and the summarized outcomes can be found in Table 6.

**Table 6:** The duration of a single cycle, measured in ms.

Scenario	GBO	AWTLS	TLS	BCRLS-MWDEKF	PSO-LSSV
#HEV1	2.0167	1.2267	1.3165	2.0867	1.207
**#HEV2**	2.0618	1.9628	1.9621	2.5628	1.28
**#HEV3**	1.8922	1.6992	1.6982	2.1261	1.192
**#HEV4**	1.8926	1.7826	1.7816	1.969	1.7326
**#HEV5**	2.1365	2.0065	2.0055	2.267	1.9065
**#EV1**	0.8406	0.6506	0.6516	0.9589	0.6211
**#EV2**	1.1656	1.0056	1.0046	1.3250	1.0011
**#EV3**	2.1538	1.1538	1.1528	1.6523	1.1438
**#EV4**	1.9962	1.6523	1.6513	1.9831	1.6323
**#EV5**	1.7260	1.3520	1.3513	1.8620	1.3220

For the HEV scenarios, the GBO algorithm performs quite well with an average cycle duration of approximately 1.98 ms. It is only slightly slower than AWTLS and TLS, which have average cycle durations of 1.72 ms. GBO’s relatively fast speed is notable, considering its accuracy compared to the other algorithms (refer to Tables 4 and 5).

For the EV scenarios, AWTLS and TLS continue to demonstrate the fastest calculation speeds with average cycle durations of approximately 1.16 ms. GBO, on the other hand, has an average cycle duration of around 1.79 ms. Although GBO’s performance is still respectable, it is slower than AWTLS and TLS in this context as well.

Overall, while GBO shows competitive performance in terms of speed calculation, there is an opportunity to enhance its efficiency further. By refining the algorithm’s approach or making optimizations, it may be possible to achieve faster calculation speeds without sacrificing accuracy. This could lead to improved performance and make GBO an even more compelling choice for hybrid electric vehicles and electric vehicles.

## 7. Limitations and future work

While our study has yielded promising results in enhancing battery state of health estimation accuracy, there are certain limitations that warrant acknowledgment. Firstly, our approach relies on specific datasets, and the generalization of our findings to diverse battery chemistries, operating conditions, road conditions, and environmental conditions may require further investigation.Secondly, the proposed algorithm’s computational complexity, while manageable, may benefit from optimization techniques to enhance real-time applicability.

In terms of future work, we intend to explore other dimensions of the suggested framework. As a starting point, we aim to verify the effectiveness of the method using other datasets related to the battery’s state of health and the driving patterns of electric vehicles and plug-in hybrid electric vehicles (EV/PHEVs). We’re also keen to investigate different types of battery chemistry.

In addition, we are keen to evaluate the algorithm’s ability to predict battery’s SOH while considering external factors such as temperature, depth of discharge and cell balancing. This can be achieved by examining battery ageing datasets that encapsulate these variables. We would also like to test the robustness of our approach under specific conditions, for example in the event of noisy input data or significant errors on one of the axes.

Our motivation also extends to improving the algorithm itself. This includes optimizing the parameters of the gradient-based optimizer (GBO) to improve accuracy and speed up the estimation process. Our aim is to find the ideal balance between accuracy and computational speed, while exploring other cost functions.

Last but not least, we are eager to implement and evaluate the framework in an on-board system. This is an essential part of our project roadmap.

## 8. Conclusion

The SOH is a strong metric to capture battery ageing level. In the present paper, a brand-new SOH estimator based on the gradient-based optimizer (GBO) is introduced. Our main objective is to improve the accuracy of SOH estimation by minimizing uncertainties in both State of Charge (SOC) estimation and measurements. To explore the robustness of the algorithm, six aggressive tests for a cell pack in HEV and EV applications were conducted. The reported methodology was evaluated against four robust techniques.

Our method delivered exceptional results across all tests. It achieves low maximum errors (0.65% to 3.21%) and mean errors (0.21% to 1.03%) in EV scenarios, and similarly low maximum errors (0.81% to 3.21%) and mean errors (0.39% to 1.03%) in HEV scenarios. These results highlight the effectiveness of GBO in accurately estimating the SOH in both EV and HEV systems. Additionally, GBO achieved low values for all predictive performance metrics, which include MSE, RMSE, and MAPE.

In the future, we plan to extend the scope of our algorithm. Specifically, we intend to evaluate the performance of GBO using additional battery aging datasets, HEV/EV driving cycles, and other battery chemistries.

- In addition, we try to continuously improve the approach byTweaking the GBO parameters to get better results.
- Speeding up the prediction, by identifying the most appropriate parameters values to obtain the most effective trade-off between precision and calculation speed.
- Examining other cost functions.

Lastly, we plan to apply and validate the proposed methodology in an embedded device.
